# Making a mark on the farm: the marks and traces of farm animals and infectious diseases in northern England

**DOI:** 10.1080/14702541.2024.2343951

**Published:** 2024-07-11

**Authors:** Niamh Mahon, Shane Finan, Lewis Holloway, Beth Clark, Amy Proctor

**Affiliations:** a School of Environmental Sciences, University of Hull, Hull, UK; b Independent Researcher, Manorhamilton, Ireland; c Centre for Rural Economy, School of Natural and Environmental Sciences, Newcastle University, Newcastle-Upon-Tyne, UK

**Keywords:** Animal geographies, artistic practice, disease, farm animals, marks and traces

## Abstract

Farmed animals are expected to move through farmed spaces in certain ways to maximise their productivity. These spaces are also designed to limit the movement of disease-causing organisms. However, both types of lifeforms do not always move in expected ways. We focus on the mark-making of these organisms to explore: 1) the evidence of their movements through farm spaces; and 2) the effects of these movements on managing farm animal disease. We explore these questions via social-scientific and artistic practices. The social science draws on in-depth interviews with UK cattle and sheep farmers, and farm advisors. The artistic component draws on work conducted by an ‘artist in residence' engaging with farm animals and farmer-livestock relationships. Farm animals and infectious micro-organisms were found to move in different ways and create different marks and traces across farms, bodies, and how diseases were managed. These lifeforms often frustrated biosecurity practices of exclusion and enclosure and existed on a spectrum of disease risk. Human actors needed to learn to become attuned to lifeform movements in order to enact disease management. We conclude by suggesting a continued focus in future social-scientific research on how the ‘sub-animal body' contributes to the enacting of farm disease management.

## Introduction

Farmed animals are expected to move through farm spaces in certain ways to maximise their productivity. Farm spaces are designed and constructed to facilitate this objective. These spaces are also designed and constructed to limit the movement of infectious, disease-causing organisms that might endanger the health and productivity of farmed animals (Hinchliffe & Bingham, 2008; Hinchliffe et al., 2013). However, both farm animals and the organisms that cause disease, such as the viruses and bacteria described by Gibbs (2022, p. 241) as ‘almost animal[s]’, do not always move in expected or intended ways. The movement of both groups of lifeforms produces evidence, in the form of marks and traces, of how they actually use these spaces. In this paper we focus on the movement and resulting mark-making of farm animals and infectious microorganisms to explore the following two questions. Firstly, what evidence, in the form of marks and traces, do these lifeforms leave as they move in and through the space of the farm. Secondly, what are the effects of these movements, marks, and traces, particularly in terms of managing farm animal disease. In so doing we address the implications of being at the limits of animal geographies through our combined focus on the marks and traces left by lifeforms, notably diseased animals that may be on the edge of life, and by paying attention to the infectious microorganisms whose admittance into the category of animals is debateable. We explore these questions via social-scientific and artistic practices, bringing two disciplinary strands of a larger interdisciplinary research project into conversation with each other, allowing us to examine this problem of being at the limits.

‘Making a mark’ has multiple meanings. Among these are mark-making as a visible feature and mark-making as producing an impression on a person, lifeform or activity. A ‘mark’ may not be imprinted on a body, but rather be a ‘trace’ left on or in a space, or in the more intangible realm of perceptions, responses and practices. We explore such marks on the places in which infectiousness is encountered on farms and consider how they can be made by infectious microorganisms on the bodies of farm animals, by farm animals themselves as they move across farmed spaces, and by humans when attempting to discover, make visible, and act upon the marks made by both infectious microorganisms and farm animals. Our paper draws on research conducted as part of the ‘Farm level Interdisciplinary approaches to Endemic Livestock Disease’ project (FIELD) (see www.field-wt.co.uk). FIELD focused on two endemic health issues affecting farm animals in the UK: bovine viral diarrhoea (BVD) in beef and dairy cattle, and lameness in cattle and sheep. Both are important because they are costly and complex to manage. They negatively affect the welfare and reduce the productivity of animals, leading to economic implications (Bennet & Ijpelaar, 2005; Mahon et al., 2021; Whatford et al., 2022). Here we focus specifically on BVD in cattle and infectious lameness in sheep, and present these as two examples of the entanglements of farmed animals, infectious microorganisms, humans, and the infrastructure and technologies on farms.

BVD is a viral disease of cattle. Viruses have been described as ‘organisms at the edge of life’ (Rybicki, 1990, p. 182) in recognition of the on-going debate as to whether they are living beings, with Fleischmann describing them as ‘beings-in-relation’ because of their need for a host to survive (Fleischmann, in press, p. 9). BVD can be passed between conspecifics via physical contact with the secretions of an infected animal (e.g. milk, semen, urine, and other bodily discharges), producing a transient infection. Transiently infected animals (TI) are temporarily ill but can recover. BVD can also be passed from an infected cow to her calf in utero, and is unique in its ability to create a ‘Persistently Infected’ animal (PI) (Houe, 1999; Moennig & Becher, 2018). PIs never recover and are infectious, shedding large volumes of infectious virus, for the rest of their lives (NADIS, n.d.-b; Nelson et al., 2016), potentially passing on BVD to other animals. Infectious lameness in sheep can have multiple causes, although in the UK most is due to bacterial infections. The latter include the diseases scald (interdigital dermatitis) and foot rot (swelling in the foot which in severe cases can lead to the separation of the hoof from the animal’s foot). These are two clinical presentations of infections caused by *Dichelobacter nodosus* (Winter & Green, 2017). Infectious lameness can also be due to Contagious Ovine Digital Dermatitis (CODD) connected to *Treponeme* bacteria (NADIS, n.d.-a). Research suggests that 90% of all lameness in sheep in the UK is caused by foot rot (O’Kane et al., 2017).

The rest of our paper is structured as follows. The next section presents a short overview of literature on human-animal relationships with a focus on BVD and infectious lameness, followed by a section exploring the literature on bodies and body parts, as well as on marks and mark-making. Next, we describe the social-scientific and artistic practices undertaken. The findings and discussion are organised into four subsections. The first three present and discuss the social-scientific work, starting with an exploration of marks on the bodies of farm animals made by and in response to disease, focusing on both BVD and infectious lameness. The second discusses the marks and traces of movements across borders and boundaries, with a focus on BVD. The final social science subsection explores the traces of movement along tracks and paths, focusing on infectious lameness. Examples of the artistic practice and output of one of the ‘artists in residence’ on the FIELD project are then presented, highlighting how the marks of disease were understood and interpreted through this approach. We conclude by reflecting on the findings and suggesting avenues for further research.

### Bodies, body parts, and mark-making

Bodies are continually in a state of becoming along with the places where they exist, and their material constitution makes a difference to how spaces are experienced (Longhurst, 2001). The interactions between bodies and the environments in which they live in, through for example touch, balance, orientation and movement, work to build a sense of place (Dixon & Straughn, 2010). Matless states that ‘life entails a becoming through environment’ (2000, p. 117). While most geographical research into place-making has focused on humans, animals have also been described as active ‘place-making agents’ (Bull & Holmberg, 2018, p. 2). In addition, the life a body lives changes the material conformation and composition of that body, e.g. via aging, adaptation to the activities in which a body participates and the challenges that a body may face, e.g. from disease (Simonsen, 2016). Thus, bodies make a difference to how an environment is encountered and experienced, and environments make a difference to the on-going constitution of bodies.

In the mid-1990s Longhurst described (human) bodies as the ‘geography closest in’ (Longhurst, 1994) and called for more of a focus in geography on the messy, visceral, corporeality of bodies. She made the case that historically bodies had been a focus of research, but they have often been approached in ways that represented them as discursive, theoretical, and fleshless (Longhurst, 2001). Later, Philo and Wilbert (2000) noted a previous lack of work that explicitly focused on animal bodies. More recent research has both explicitly acknowledged the porous, leaky, and entangled nature of bodies (Lisle, 2021) and taken a greater interest in animal bodies. Geiger and Hovorka (2015), for example, explore what it means to be a donkey in southern Africa, considering how donkey identities are co-constructed by their relationships with humans and the environment in which they dwell. Fox et al. (2023), meanwhile, explore how both humans and dogs are transformed via the physical and sensory dimensions of canine training classes. They make the case that when humans and animals live and work together, they become attuned to each other and take part in cross-species, embodied communication. This is not always positive, however, and misunderstandings and frustrations can and do occur (also Laurier & Arathoon, this issue). There has been some work on bodies in relation to farm animals, especially around breeding farm animals for particular purposes. Examples include work by Yarwood and Evans (2006) on the role of breed societies in shaping the geographies of certain Welsh sheep breeds, and Holloway and Morris (Holloway, 2005; Holloway & Morris, 2014) on the genetic and visual evaluation of the bodily composition of pedigree cattle and sheep.

Related to the above is literature addressing the materials that make up a body (Dixon, 2014; Smelik & Lykke, 2008), particularly the fleshy, messy parts of bodies and the visceral processes occurring within bodies, described by Squire (2000, p. 6) as the ‘sub-animal body’. The sub-animal body has also been engaged in creative works. Christien Meindertsma’s piece ‘Pig 05049’ (Meindertsma, 2007) explores the fragmented afterlife of a single pig (the eponymous 05049) and how different parts of 05049s body are used as raw materials in a wide range of products, including more familiar items, such as wine and cheesecake, and ones that might be considered as more unexpected, such as ammunition and sandpaper (Vanden Eynde, 2021). Smelik and Lykke (2008, p. ix) describe these parts of living things as ‘the bits of life’, since here body is not the final, or smallest, unit worthy of analysis because there is explicit acknowledgement that body parts may exist and be thought about *apart* from the organism from which they originated (Dickinson & Johnson, 2022). We draw on these literatures to focus on farm animals and the infectious microorganisms that cause disease, taking an interest in how diseases mark farm animal bodies and body parts, as well as in how bodies are marked in response to disease and the ways whereby marked bodies may play a role in the co-production of infectious farm animal disease.

As noted, traces are a particular subset of marks related to movement, described by Ingold as ‘any enduring mark left in or on a solid surface by a continuous movement’ (2016, p. 43). They can be used by humans to become attuned to the difference animals make to a situation (Dempsey, 2010). One such trace is the material evidence of an animal’s movement, such as the paths across a field created over time by recurrent trampling. Lorimer’s descriptions of the marks made and used by reindeer moving across a landscape are an example: these animals use and understand space by repeatedly moving through it – ‘a herd knows this geography though its many moving feet’ (Lorimer, 2006, p. 498) – and in the process they leave tangible traces of their movement. Thus, landscapes are ‘not only ours’ (that is, made by and belonging to humans) and by attending to the traces of movements these spaces can be described and understood in non-human ways. Unlike wild animals, farmed animals are expected to move through farm spaces in certain ways and farm spaces are constructed to facilitate regimented movements and limit unexpected ones (Bächi, 2016; Netz, 2004).

In moving in expected ways Despret notes the following about dairy cows participating in the milking process: ‘when they do what they must so that everything happens as it is supposed to, we do not see this as testimony to their willingness to do what is expected of them. … [T]heir obedience looks mechanical’ (Despret, 2013, pp. 42–43). Thus, instead of seeing the cows’ active participation in farming activities, Despret suggests that a human observer might instead interpret these actions as evidence of the mindlessness of cattle (Fudge, 2017). In moving in intended ways, the work performed by the cows (Porcher & Schmitt, 2012), and the choice that the cows may be making to participate in the work of being milked, becomes invisible. Yet animals do not always move in expected or intended ways, with Porcher and Schmitt (2012), Hansen (2014) and Holloway et al. (2014) giving examples in relation to dairy cows. Philo and Wilbert (2000:, p. 14) note that ‘[animals] often end up evading the places to which humans seek to allot them’, leading such animals to become out of place, transgressing human spatial orderings (Matless, 2000) and notions of where they should be (Cresswell, 1996). By moving in unintended or unexpected ways, the work that farm animals usually do – and may now not be doing – becomes visible. These acts can be seen in certain situations as a form of transgression, as when moving to cross a border or boundary. Transgression does not ‘rest on the intentions of actors, but on the results – on the “being noticed” of a particular action’ (Cresswell, 1996, p. 23). Whether an animal, particularly a farm animal, has transgressed in a space depends on the perspective of a human and on evidence of the movement being noticed by humans. Matless (2000) illustrates this via the example of coypu in the UK (a South American animal introduced to the UK via escapees from fur farms), which were seen by some as a welcome addition to the ecology of wetland regions and by others as invasive and transgressive, in need of eradication. This matter is further complicated when considering that animals may act as vectors for infectious microorganisms, and that these may also disrupt human spatial orderings (Fleischmann, in press; Halfacree & Williams, 2021). For example, a farm animal’s movement may facilitate a microorganism’s transgression across a boundary, even if the animal itself is not transgressing by performing this action**.**

Although this paper focuses on the marks of disease across space, it is worth noting that there are temporal dimensions to the management of farm animal health (e.g. work by Enticott & O’Mahoney, 2024). As mentioned previously, bodies are in a constant state of becoming, and the life a body lives changes the conformation and composition of that body (Simonsen, 2016). Furthermore, the marks and traces of disease emerge, change, and possibly fade over time and across different time scales. Farmers need to become attentive to the temporal as well as spatial changes in disease, which can be perceived in different ways, e.g. through sights, smells, and changing animal behaviours.

We bring together the ideas discussed above to explore the movement and resulting mark-making of farm animals and infectious microorganisms, seeking to uncover the impact that these movements have on the enactment of disease management. We draw on in-depth social-scientific and artistic research with farmers, farm advisors and farm animals in the north of England to explore these concerns. In the next section we describe the different approaches and methods taken to explore these questions.

### Relationships between farmers, farm animals and infectious disease

Both BVD in cattle and infectious lameness in sheep are examples of ‘production conditions,’ described as such because they are associated with certain ways of farming animals and particular sets of on-farm relationships between humans and farm animals, and also between farm animals and other non-human animals (Holloway et al., 2023a; Nir, 2003). Production conditions impact both the welfare and productivity of farm animals (Holloway et al., 2023b, 2023c), and the incidence and severity of these conditions is impacted by the particular management practices, physical environments and on-farm relationships experienced.

The management practices employed and the degree of physical contact between cattle are key to the spread and severity of a BVD outbreak. Within housed systems, a PI calf can infect more than 90% of the herd by the time it is 4 months old. In contrast, when cattle are kept in separate pens or different buildings, BVD transmission is delayed (Houe, 1999). Herd size can also impact the severity and longevity of an outbreak. Total immunity via the transient infection of all animals in a herd is more likely to occur in smaller herds, where there is a greater opportunity for the animals to be in contact with each other and therefore with any infected animals. Total immunity is less likely to occur in larger herds, where the opportunity for all animals to mix with each other is more difficult to achieve (Moennig & Becher, 2018). At the regional scale, it has been noted that areas with higher densities of cattle have a higher prevalence of BVD infection than those with lower densities (Houe, 1999).

Climate, on-farm management practices, and animal characteristics all influence the spread and severity of foot rot in sheep. The temperate, damp conditions found in the UK are optimal for the reproduction and spread of the disease (NADIS, N.D.-a), with *Dichelobacter nodosus* able to persist in damp soil up to 24 days (Zanolari et al., 2021). Management practices influence the spread of foot rot, with high stocking densities (Busin, 2018), the introduction of infected sheep to a flock, maybe through trade (Zanolari et al., 2021), and the use of foot trimming and foot bathing – traditional management practices for lameness control (Laven, 2017; Winter & Green, 2017) – all contributing to the spread and persistence of infectious lameness. The genetic characteristics of sheep also play a part, with some breeds, such as the Romney Marsh, being more resistant than others, such as Merino sheep (Storms et al., 2022). Some authors (e.g. Bellet et al., 2021) have noted that the current management of chronic farm animal health conditions focuses on reactive treatment and attempting to breed more resilient animals, rather than addressing the problems inherent in the systems within which these animals exist.

There are many practices – often cast as ones of ‘biosecurity’ – that aim to stop the spread of disease (Bingham et al., 2008; Hinchliffe & Ward, 2014; Shortall et al., 2018). Enacting on-farm biosecurity requires care for farm animals. Care is complex, ambiguous, and associated with obligation and responsibility (Tronto, 2020). It requires close relationships and an anticipation of need (Brown & Dilley, 2012; Lawson, 2007). For farm animals, this means acknowledging that their ‘use’ affects them and responding appropriately (Holloway et al., 2023a). Wilkie (2005, 2010) inquires into how farm animals exist in northern UK farming systems as both commodities and sentient beings (Wilkie, 2005; 2010). The status of these animals is dynamic and depends on the role to which they are assigned within the production system, particularly whether an animal is kept for breeding or for fattening and slaughter. When an animal deviates from what is expected of it, such as changing in status to being a diseased animal, it stands out from the group. The animal can thus become recognised as an individual by the human(s) working with it, and the relationship between the two may change to one of increased attachment and decommodification. Moreover, endemic health conditions on farms in northern England can often become normalised, for both BVD and lameness, remaining present but unseen by farmers, and subsequently farmer advisors such as vets must work hard to reveal the problems here to farmers (Holloway et al., 2023b).

## Studying mark-making: social scientific and artistic approaches

The social-scientific elements of this paper draw on in-depth interviews conducted with cattle (both dairy and beef) and sheep farmers (*n* = 29), and farm advisors (*n* = 21), in northern England (see Table 1 and Table 2). The farmers were selected to illustrate a range of farm types and environments, including upland and lowland locations and larger and smaller farms. Farm advisors were chosen because of their geographic location and/or their experience and knowledge of cattle and sheep and the two diseases under investigation. Interviews explored the interviewee’s background and farming history, their knowledge and experience of BVD and lameness, and how they acquired and used knowledge and information. Interviews were audio-recorded and transcribed verbatim. The transcripts were coded using Nvivo software (QSR International Pty Ltd., 2020), using a codebook developed by NM, BC, LH and AP.

**Table 1.** Farmer interviewees.

Interview ID	Interview type	Gender	Livestock mix	Upland or Lowland
.Farmer 1.	.Farmer.	.Female.	.Sheep.	.Lowland.
.Farmer 2.	.Farmer.	.Male.	.Dairy cattle.	.Lowland.
.Farmer 3.	.Farmer.	.Female.	.Beef cattle and sheep.	.Upland.
.Farmer 4.	.Farmer.	.Male.	.Beef cattle and sheep.	.Upland.
.Farmer 5.	.Farmer.	.Male.	.Beef cattle.	.Lowland.
.Farmer 6.	.Farmer.	.Female.	.Beef cattle and sheep.	.Upland.
.Farmer 7.	.Farmer.	.Male.	.Beef and dairy cattle, sheep.	.Upland.
.Farmer 8.	.Farmer.	.Male.	.Beef cattle and sheep.	.Upland.
.Farmer 9.	.Farmer.	.Female.	.Beef cattle and sheep.	.Lowland.
.Farmer 10.	.Farmer.	.Male.	.Beef cattle and sheep.	.Lowland.
.Farmer 11.	.Farmer.	.Female.	.Sheep.	.Upland.
.Farmer 12.	.Farmer.	.Male.	.Beef cattle and sheep.	.Upland.
.Farmer 13.	.Farmer.	.Male.	.Beef cattle.	.Upland.
.Farmer 14.	.Farmer.	.Male.	.Beef cattle and sheep.	.Upland.
.Farmer 15.	.Farmer.	.Male.	.Dairy cattle.	.Upland.
.Farmer 16.	.Farmer.	.Male.	.Dairy sheep.	.Upland.
.Farmer 17.	.Farmer.	.Male.	.Dairy cattle.	.Upland.
.Farmer 18.	.Farmer.	.Male.	.Dairy cattle.	.Upland.
.Farmer 19.	.Farmer.	.Male.	.Dairy cattle.	.Upland.
.Farmer 20.	.Farmer.	.Male.	.Beef cattle and sheep.	.Upland.
.Farmer 21.	.Farmer.	.Male.	.Beef cattle and sheep.	.Lowland.
.Farmer 22.	.Farmer.	.Male.	.Beef cattle and sheep.	.Upland.
.Farmer 23.	.Farmer.	.Male.	.Beef cattle and sheep.	.Upland.
.Farmer 24.	.Farmer.	.Female.	.Beef cattle and sheep.	.Upland.
.Farmer 25.	.Farmer.	.Male.	.Beef cattle.	.Lowland.
.Farmer 26.	.Farmer.	.Female.	.Sheep.	.Lowland.
.Farmer 27.	.Farmer.	.Male.	.Beef cattle and sheep.	.Upland.
.Farmer 28.	.Farmer.	.Male.	.Sheep.	.Upland.
.Farmer 29.	.Farmer.	.Male.	.Beef cattle and sheep.	.Upland.

**Table 2.** Adviser interviewees.

Interview ID	Interview type	Gender	Advisor type
.Advisor 1.	.Advisor.	.Female.	.Pharmaceutical representative.
.Advisor 2.	.Advisor.	.Male.	.Cattle hoof trimmer.
.Advisor 3.	.Advisor.	.Male.	.Cattle hoof trimmer.
.Advisor 4.	.Advisor.	.Female.	.Veterinary consultant.
.Advisor 5.	.Advisor.	.Female.	.Levy board staff.
.Advisor 6.	.Advisor.	.Female.	.Livestock nutritionist.
.Advisor 7.	.Advisor.	.Female.	.Vet.
.Advisor 8.	.Advisor.	.Male.	.Vet.
.Advisor 9.	.Advisor.	.Male.	.Veterinary consultant.
.Advisor 10.	.Advisor.	.male.	.Vet.
.Advisor 11.	.Advisor.	.Female.	.Vet.
.Advisor 12.	.Advisor.	.Female.	.Vet.
.Advisor 13.	.Advisor.	.Male.	.Farm consultant.
.Advisor 14.	.Advisor.	.Male.	.Veterinary consultant.
.Advisor 15.	.Advisor.	.male.	.Livestock auctioneer.
.Advisor 16.	.Advisor.	.Female.	.Vet.
.Advisor 17.	.Advisor.	.Female.	.Farm consultant.
.Advisor 18.	.Advisor.	.Female.	.Farm consultant.
.Advisor 19.	.Advisor.	.Female.	.Assurance scheme assessor.
.Advisor 20.	.Advisor.	.Male.	.Vet.
.Advisor Group.	.Advisor.	.Female & Male.	.Advisory services for vets.

As part of the FIELD project three artists-in-residence (AiR) – Mark Jones, Michele Allen, and Shane Finan (SF)^1^ – were commissioned to create works facilitating inclusive, reflective, and non-judgemental exchanges between farmers and members of the non-farming public. Due to Covid-19 restrictions, the AiR resided virtually with the FIELD researchers and in-person with different UK farming communities to undertake creative practices. The AiRs’ work was informed by that of the researchers and sought to engage with dimensions of farm animal health and welfare and farmer-livestock relationships. The residencies enabled the formation of place-based multispecies contact zones between artists, farmers, and farm animals (Woods, 2023).

SF approached the commission via an understanding that the embodied experience of a sheep or a cow is something that we as humans cannot have. To try to do so would be to identify with something that has a body altogether different from ‘our’ (human) own. However, there are ways to experiment with understanding the embodied experience of a nonhuman organism, particularly via creative practice. One suggested methodology from design ethnographer Anne Galloway is to use a process of being-with, spending time with nonhumans, to allow an exchange of experience (Choi & Galloway, 2021), deepening understanding through emotional action and reaction. The emphasis is more empathetic than scientific, but has been seen to have observational merit, such as in the research of Jocelyn Porcher (Porcher, 2017). Understanding embodiment is a muddy research process that cannot provide conventional evidence, but rather offers experience. For example, Thomas Thwaites designed goat-like legs to live among goats, on all fours, as an experiential research project, and presented this embodiment as documentation in the project *Goatman*^2^ (Thwaites, 2016). SF drew from these works when undertaking his artistic practice.

Examples of SF’s art are presented in this paper. All images are presented with SF’s consent and photographs of farmed spaces are presented with the consent of the farmer. These pieces work to illuminate facets of animal movement and mark-making that have been engaged with in different ways to the social-scientific researchers. The pieces are not intended to be an add-on to the social scientific findings. They stand both on their own and in conversation with the social science. In the section that follows, we present the findings and discussion in four subsections: the first three focussing on the social science and the fourth on the artistic practice and outputs.

## Exploring the marks of animals and infectious diseases

### Bodies: marks and traces from and in response to infectious disease

This subsection explores the marks made by infectious microorganisms on the bodies of farm animals, and the requirement for humans to be attentive to these marks when managing the health of farm animals. This management may involve further mark-making by humans on animals’ bodies in response to disease. Infectious microorganisms can mark the bodies of animals they infect. For certain diseases these marks are unique – the smell of foot rot distinguishes it from other types of infectious lameness in sheep – but for others the marks can be non-specific, indicating that the infected animal is in poor health but not what the cause is. The marks of disease can therefore be more or less identifiable to humans. This is the case for many of the symptoms of BVD (NADIS, N.D.-b). Interviewees mentioned the hidden nature of BVD as making it particularly difficult to manage:

… you realise that it [BVD] can be there, but you don't know you've got it, and you could be spreading it instead of stopping it … . (Farmer 24)

… it’s a hidden disease. Well, it doesn’t always show. It takes long to incubate, to manifest its symptoms. (Farmer 27)

This means that farmers must use other identification methods, such as ear tag testing, described as ‘where we take a little circle of tissue out [of the ear] and get it tested’ (Farmer 15), to make the presence or absence of infectious microorganism visible. This further marks the bodies of animals physically: the removal of ear tissue creates a mark on/in the body of the animal. However, the mark can also be more intangible, even metaphysical. To extend Ingold’s concept of the ‘ghostly line’ (2016, p. 47), the results of the ear tag testing facilitate the animal’s movement across a health-related ghostly line. According to Ingold, ‘ghostly lines’ are those marks and traces without a physical presence in the environment, with examples including the lines between stars that make up constellations, time zones, and lines of longitude and latitude. In the case presented here the animal tested for BVD moves across a ghostly line that demarks its disease status once the ear tag test results are known to the farmer. Thus, before the results are received, the animal’s disease status is unknown or may be assumed to be negative. Yet, once the farmer receives the results, the animal moves across the line from an unknown to a known disease status and is subsequently marked as such in farm records. This mark has implications for the life of the animal: it may be rendered killable and culled (Holloway et al., 2023a), or it may be treated for BVD and recover.

The different physical marks made by infectious microorganisms on farm animal bodies mean that farmers and advisors must become attuned to the visceral and sensory cues of disease and the messiness of diseased bodies (Longhurst, 2001). The excerpt below illustrates this point in relation to infectious lameness in sheep:

… when you’ve got foot rot, the foot rot has gone in underneath the hard hoof. It makes it go white, pussy and horrible and the hoof starts to break off from the actual bone, I suppose, or the actual sheep. (Farmer 7)

Here a farmer discusses the actions of the infectious microorganism as it moves and marks the sheep body. Farmers also discussed learning to become attentive to the differences in the marks made by the different species of microorganisms, as illustrated by the following quotation:

Foot rot is between a thing [the hoof], whereas CODD is generally further up [the hoof]. You can tell … There are very many presentations that show the lovely differences, it is quite obvious. (Farmer 6)

Marks caused by infectious microorganisms may also differ in whether it is the whole body or parts of the body being marked. BVD, when it is seen, may mark the whole body, for example by creating ‘poor, ill-thrifty cows’ calves that aren’t growing well’ (Advisor 16) and ‘a calf that is just a bit on the light side and isn’t doing that well … I guess they’d call it a “poor doer”.’ (Advisor 5). In contrast, infectious lameness, as discussed by Farmers 6 and 7 above, presents on certain body parts of the animal, notably parts of the hoof.

Marks also move and change in space and time. Infectious diseases can become progressively worse, producing more or different marks: the animal can recover, and the marks may fade, or they may linger post-infection. These relationships become more complex when the initial infection facilitates the entry of other microorganisms, causing secondary infections. For BVD, the initial infection might be hidden, as discussed previously, but secondary infections may be more visible. This is also the case for CODD, a cause of infectious lameness in sheep, in which secondary infections can be identified via the sense of smell, as indicated here:

Interviewee: CODD, it’s an awful disease and it’s much harder to treat and it’s much harder to eradicate … It’s a horrible thing. You can get complete, where the hoof will drop off in severe cases, it really is a dreadful disease, and it smells something terrible as well.

Interviewer: I’ve heard of foot rot smelling but I haven't heard of CODD smelling, as well?

Interviewee: I think it’s a secondary infection because I’ve read about it as well, some people say: ‘CODD doesn’t smell,’ well, it certainly does, but I think it’s the secondary infection that has that horrible smell. In a bad case of CODD, even in the field, if you gather a flock of sheep, it has a smell of its own that I can actually recognise. (Farmer 28)

Again, this excerpt highlights the interviewees’ engagements with the visceral nature of diseased bodies.

When the disease is ‘perceived’ (seen, smelled, felt, tested for), farmers and advisors can work to treat the infection. This treatment can itself leave further marks on the bodies of farm animals. Below, a farmer describes actions taken to treat infectious lameness and how this marks sheep bodies:

Often by the time you thin [the hoof], by hand you could just peel the bloody claw off, it’s the whole outside and the flat bit that it walks on just comes away. It’s generally just fastened on underneath where the hair stops, between the hair and the hoof, and you just trim that off and that all pretty much comes away. It’s usually just on one side, one claw, so it has the other claw to stand on and within a few weeks it grows new … it dries up, because you’ve let air at the sore red bit, it’ll scab over, and you put Terramycin spray on and a jab. But letting air at it must dry it up and kill the bugs, they can’t live because they must be in the open and they haven’t got the shelter of the nice warm hoof sticking on top of them. Once you take the skin off, they just seem to heal up pretty fast and they’re away. (Farmer 7)

Here the marking of the animal includes the use of Terramycin spray, an antibiotic aerosol treatment which contains blue dye to show where treatment has been given, providing another example of mark making. Farmers also mark animals already marked by disease. This makes an animal easier to find for subsequent treatment, or removal from the group, rather than as a method of treatment. The following excerpt relates to the management of lame sheep:

… we have a system; we’d colour spray mark their ears when we inject them with an antibiotic. Where a sheep comes in and its lame, we’ll turn it over, look at its feet, see what’s wrong, sort it out. If it needs an injection, we’ll give it an injection and we’ll spray its ear red or something like that depending on which antibiotic it is. We’ll spray the right ear first and then if they come in again another time and you have to do it again then you spray the left ear, so you know which sheep aren’t getting right when you’re treating them. When we’re getting rid, at the back end, when we get rid of our sheep that we don’t want, they go in with the sheep that we don’t want. So, we sell them fat usually and we also have another system if we know something has a problem, any kind of problem that it needs culling for, then we put a bright pink tag in its ear, so we can see which they are quite easily. (Farmer 12)

In these cases, marking makes an animal known to be diseased, or particularly susceptible to disease and therefore a risk to other animals’ health and productivity, obviously visible. Thus, the life and health of the individual animal is weighed against the life and health of the herd or flock of which it is a part (Holloway et al., 2023a).

### Borders and boundaries: BVD and bovine bodies

Humans expect farm animals to stay within the boundary of a farm, often employing physical structures such as fences and walls to divide space, limiting animal movements (Netz, 2004) and marking space as either within or without the farm. Within a farm, farm animals are expected to stay in a particular space, unless moved by a human for a particular reason. However, this is not always the case and farm animals can and do cross barriers and boundaries, overcoming limits to their movement. In doing so they may unintentionally facilitate the simultaneous movement of infectious microorganisms, destabilising human-made borders (Fleischmann, in press). These events create marks and result in challenges to the management of infectious disease. This subsection explores movements across borders and boundaries and the marks made by them, focusing on cattle and BVD.

Cattle movements were often thought of by interviewees in terms of degrees of disease risk. This was modulated by various considerations, including which boundaries are crossed, and which animals are crossing them. In the following comment a cattle farmer discussed the risk of BVD to their own animals posed by someone else’s cattle crossing the boundary between the farms:

I've always got the risk of naïve young stock coming into contact with somebody else's cattle. Say, somebody else's cattle break down the fences and get through. (Farmer 21)

Here a mark of the movement would be the destruction of the fences that act as a physical border between the farms, the trampling around the broken fence, and the cattle becoming infected with BVD. In this excerpt the interviewee acknowledges the nature of animals: their unpredictability, especially if unknown (those belonging to the neighbouring farm). Farmer 21 suggests that unknown animals might transgress, but does not mention their own cattle transgressing, which may be because they are known and therefore more predictable. Furthermore, the risk of BVD is mentioned in relation to a particular group of cattle, the ‘naïve young stock’, where ‘naïve’ is used in a clinical sense to refer to animals that have not been exposed to BVD via a transient infection or vaccination. Thus, BVD is particularly risky for these young, naïve animals, rather than all the cattle on the farm, the riskiness of the encounter amplified by these cattle having never before encountered the infectious microorganisms.

When mentioning reasons why cattle might cross a boundary, interviewees often brought up unsanctioned mating events, which also pose a disease risk. Animals are not only crossing a boundary, but also potentially creating calves as a mark of this movement. Two excerpts are presented below: in the first the mating is unsanctioned, but not unwelcome; in the second it is both unsanctioned and unwelcome, producing a PI calf.

[O]ur bull jumped over the fence into one of the other neighbours and was running around with his cows. He didn’t ring me up or anything. I saw him in the pub a few days later and he said, ‘The bull alright?’ I said, ‘Yes, he’s fine.’ ‘Oh good.’ I said, ‘Oh right. Has he been over the fence?’ He said, ‘Yes, it’s not a problem. He’s a cracking bull. I just want to know what his health status is.’ He was pedigree Shorthorn. He had come from [redacted] so he was off the high health scheme. He [the other farmer] was just happy with that. (Farmer 23)

I do know someone else that also struggles with BVD, and it was because a neighbour’s bull jumped the fence and got in with some of her heifers and it was a while before she could get it out again, and it was obviously nine months^3^ after then that she found out she’d got a PI. (Farmer 9)

The difference between the two movements was the presence of BVD travelling along with the bull across the boundary. In the first case the lack of a microorganism ‘passenger,’ which would be invisible to the human eye, is made visible by its absence through the farmer’s membership of a ‘high health scheme’ (an industry accreditation programme requiring farmers to eradicate diseases such as BVD from their herds). The invisibility of BVD – often referred to as a hidden disease (an idea which we will revisit in the next subsection, which focuses on infectious lameness) means that farmers must use other identification methods to make the microorganisms visible, in this case by testing for BVD, which provides a positive or negative mark in the form of a laboratory result or membership of the high health scheme. Interviewees also recognised the intra-species relationships between the cattle. The bull’s movement may disrupt human desires (to maintain a division between the different herds, in part because of biosecurity considerations), but the fences disrupt the bull’s desires (to move and to mate) and relationships between the cattle from different farms. These movements can be seen as a form of ‘relational resistance’ (Bear & Holloway, 2019) that emerges from the relationship between the cattle, humans and technologies employed to limit animal movements.

For BVD to transgress, the whole animal infected with the disease does not have to cross the boundary. Instead, just part of an animal needs to cross to facilitate the movement of the virus from one animal to another, as illustrated by the following quotation from a farm advisor:

The majority of our farmers can’t not have boundary contact. And most of it is stone walls, to be fair, within a barb [barbed wire fence] so there is a break between them. But nose to nose would still be possible. (Advisor 7)

Nose-to-nose contact between cattle is a well-known route for BVD to spread, with research suggesting that one hour of contact between an infected and an uninfected animal is enough to spread the disease, with PI animals more effective spreaders of the disease than TI animals (Houe, 1999). In the quotation above, the physical touch between the body parts of the two animals destabilises the boundaries that separate farms, herds of animals and potentially diseased from non-diseased life (Dixon & Straughn, 2010). The nose-to-nose contact, and the subsequent movement of nasal secretions containing the BVD virus from one nose to another, facilitates the spread of infectious microorganisms and highlights the role of the sub-animal body in the spread of disease (Dixon, 2014).

This subsection has illustrated how animals, both whole and in parts, may move in unintended ways across farms, crossing boundaries and producing traces of their movements. Often a mark of this movement, and what makes it transgressive in the eyes of the farmers and advisors, is the observed result of the movement: for example, broken fences and/or animals in the ‘wrong’ places. The observed result is the spread of infectious disease. Common to all the quotations is a consideration of which boundaries are being crossed. Moving between different farms was seen as particularly noteworthy because of the unknown disease status of the other farm, and the other animals. In the next subsection we explore the marks made by animals within and across the surface of the farm itself.

### Tracks and paths: sheep in motion

This subsection explores the traces – the subset of marks related to movement – made by the movement of farm animals across farmed spaces via tracks and paths, with specific reference to sheep and infectious lameness. In repeatedly moving across a field along a certain route sheep create traces in the form of pathways. Other sheep follow the pathways, responding to the traces made by other animals. In the excerpt below pathways that sheep make in long grass reduces the risk of the spread of infectious nematode ‘worms’, but increases the risk of infectious lameness:

So, although there’s advantages in having long grass, I have less worms, the sheep tend to form a track in it and therefore they transmit the lameness. (Farmer 27)

Here the farmer is aware of typical sheep behaviour, how they choose to use the spaces they occupy and how this may increase the risk of lameness. Sheep are flock animals that tend to move as groups, follow each other and in doing so make and use pathways (Armstrong, 2018). The farmer uses this knowledge – and that in doing so the animals facilitate the movement of infectious lameness, to predict the movement and spread of disease-causing lifeforms. Here the lifeforms are both micro – (the bacteria that cause infectious lameness) and macro – (the worms that parasitise sheep) organisms. The farmer can subsequently alter their disease management practices. In the next example, the paths taken by the animals across a watercourse in a particular field reduces their risk of lameness:

The cattle and the sheep are crossing these watercourses all of the time, a lot of them cut through the middle of fields, this way and that way, and sheep and cattle are crossing over them all the time. So, they are getting their feet washed, so I don't know whether that … but we can fetch lame sheep from down the road back up to here and dress them and they are fine within a few weeks, you dress them down there and they never seem to get better, constantly doing them … it must be something in the land.’ (Farmer 7)

In both examples, the farmers have become attentive to how the animals use their space and that this makes a difference to managing infectious disease. The farmers in these examples deploy their knowledge about both the animals’ behaviour and the space in which the animals live, echoing the reindeer herders described by Lorimer who ‘learn to think like a reindeer’ (Lorimer, 2006, p. 502).

The excerpts above illustrate how farmers can be attentive to the marks that animals make on the farm landscapes. This knowledge can make a difference to the farming practices undertaken to manage infectious disease. The following excerpts illustrate how the manner in which sheep choose to move through different farm spaces can make an impression – a type of mark – on how a farming practice is (or is not) performed:

We used to have one [footbath] where we could put them in and shut them in, so they would have to stay in for as long as we wanted them too in a way, but we had to get rid of it because pushing them in was just a nightmare. (Farmer 1)

I tried once or twice in the shed to put one of those pads down near the water trough with zinc sulphate in it and they would then stand in there for a while, but that didn’t work so well because you had train them to go through the gate to drink the water … It took too long to train them, so it didn’t really work very well. (Farmer 27)

The farm animals are not passive in these situations; their being there makes a difference to what is or is not possible: in the first excerpt the sheep did not move to the correct place, in the second they did not stay in place long enough for the technology to be effective, and in both cases the farmers did not want to work to move or train the sheep to perform these tasks. Thus, these are further examples of that ‘relational resistance’ mentioned earlier, wherein the farmers are not fully in control of the situations. There is hence as emphasis on the kinds of situated negotiations between farmers, sheep and the technologies employed (the footbaths and medicated pads) that occur when managing infectious lameness.

### Artistic outputs: bodies in motion

In this subsection we present examples of the art produced by SF in relation to the marks and traces made by farm animal movements, and particularly the bodies of sheep with lameness. As an AiR on the FIELD project, SF spent time with sheep with lameness, developing an understanding of the physical implications of the different afflictions that cause lameness by closely observing and mimicking the movements of these sheep. SF began to understand how the feet of lame animals, led to limping, but that the visibility of embodiment of this limp, the mark it makes, is not always obviously in the feet. Rather, the movement of the spine, while compensating for one or more afflicted feet, creates a bob in the head of a sheep while it moves. When a sheep is lame, this leads to different flock behaviours, and SF observed that lame sheep keep more distance from a visiting human than those that are not lame. This echoes observations by primatologist Thelma Rowell, published in a paper by Despret (2006). Rowell had spent her life documenting the behaviour of primates. When she returned to Yorkshire to leave this career, she observed sheep, applying the same methods of analysis. She noted that a sheep with a shepherd nearby acts more brazenly than without, because the sheep knows the shepherd is both predator and protector.

**Figure 1.**
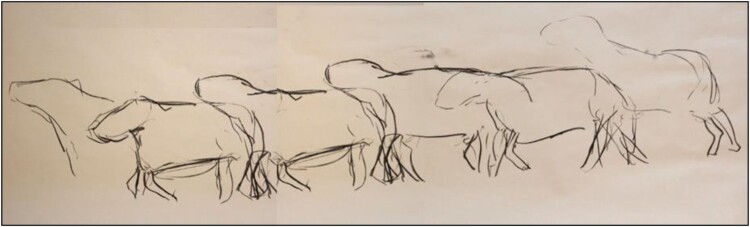
‘Lameness movement’ (Finan, 2020a).

**Figure 2.**
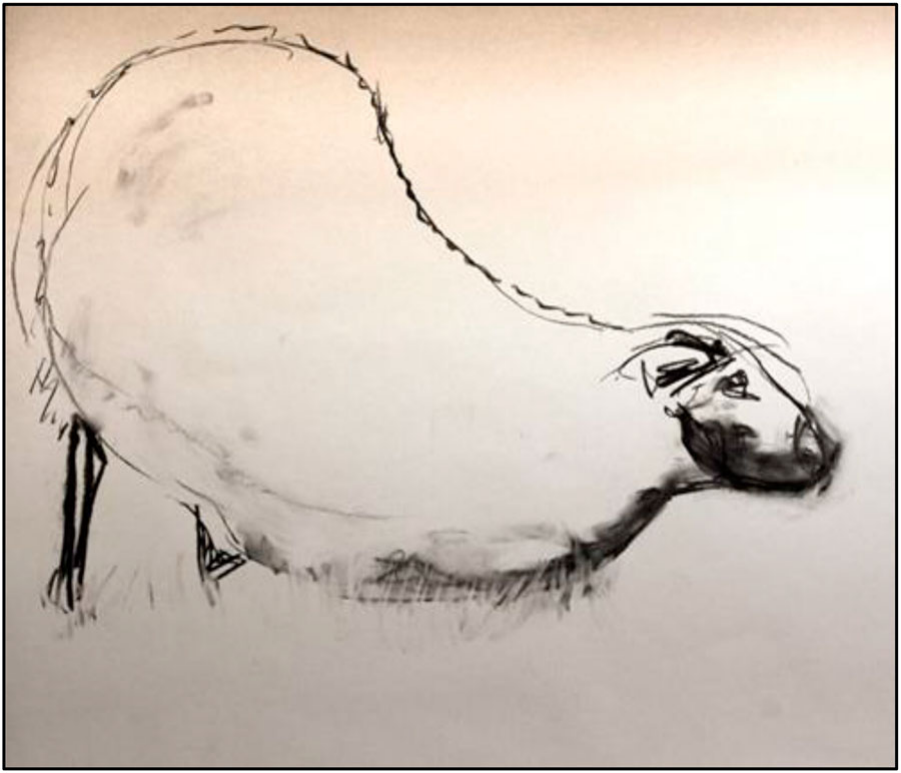
‘Dandelion kneeling’ (Finan, 2020b).

**Figure 3.**
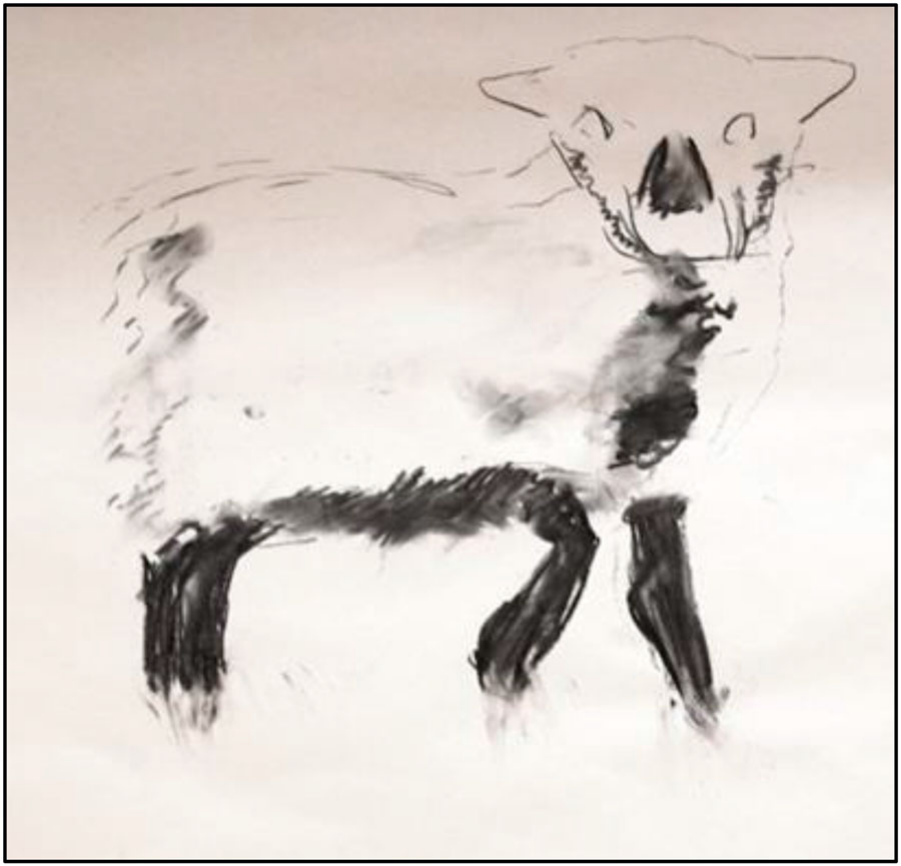
‘Lamb leg’ (Finan, 2020c).

SF’s work explored the visible effects, marks and traces of lameness on farms. He visited nine farms in Scotland, England and Ireland over six months, and spent time with lame sheep. While on site, he took photographs, made videos and drawings and text responses to what he witnessed (see Figures 1–3). He learned from individual sheep, following their movements and positions, noting how their heads moved when they limped or how much longer they rested or kneeled when they were struggling. The aim was to find what is often invisible or unseen in lameness, and how it marks the movement of the animals. As an observer in a field, SF was aware that his presence changed the animals’ behaviours. This has parallels to the example presented by Fox et al. (2023) of the transformational nature of human-dog relationships during canine training classes. In taking part in prolonged, close observation, both SF and the flock were transformed by the encounter. Here, this transformation manifests as a change in sheep behaviour and in SF gaining a better understanding of the movements and behaviours of vulnerable, lame sheep. How humans become attentive to the physical and behavioural marks of disease in and on farm animals is a theme of course threaded through the social-scientific contributions to this paper.

**Figure 4.**
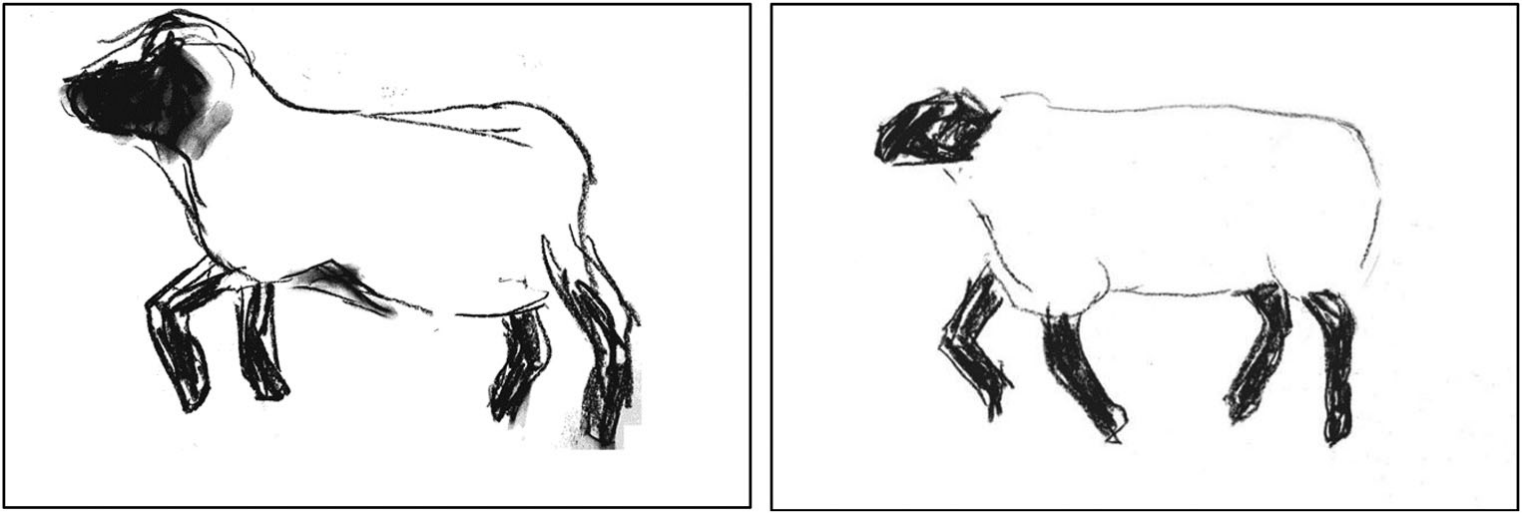
Screenshots from an animated gif illustrating the movement of a lame sheep (Finan, 2020d).

As SF brought together his the research, he began to create collaborative online artistic responses that included written diary entries, audio, interactive games, image, and video. These composites were designed to consider at a distance the visible in lameness and in human-animal interactions. For example, from experience of having sheep cautiously approach once they were used to him, one piece features an animated sheep that will only approach on-screen when a visitor stays still (i.e. does not move the computer mouse), while elsewhere the animated sheep run from the human body whenever it moves (Finan, 2020d). Another section features an animated gif of a sheep in motion^4^ (see Figure 4), reflecting the head bob, as a mark of lameness, noted above.

**Figure 5.**
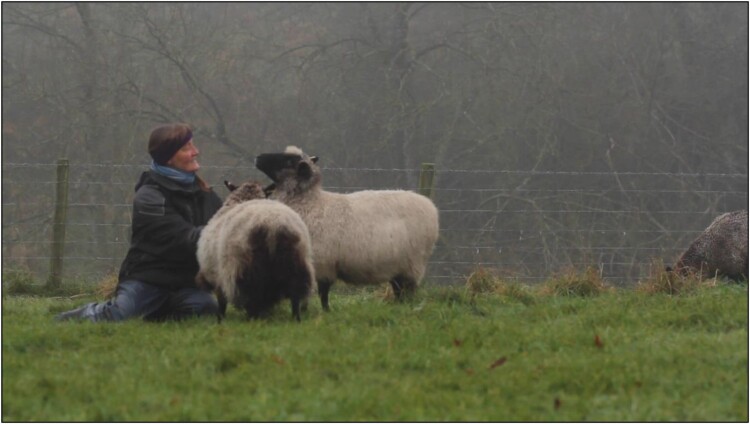
Still from video by Shane Finan: Farmer F was approached by her flock almost immediately once she had moved away from SF. One sheep suffering lameness kept a large distance from SF but came to F (not pictured, recorded in notes).

Learning from the methodologies of ethnographer Galloway, designer Thwaites, and artist Lynch (outlined in the methods section), the process of being-with as a way of understanding both through observation and through bodily enaction became the research method. The processes involved sensing and sense-making with nonhumans, co-observing through a process that Livio describes thusly: ‘sensing and making sense through nonhuman beings in research requires acknowledgment of those beings as knowledge coproducers’ (Livio, 2022, p. 237). While an engagement between human and nonhuman that takes place in unfamiliar settings over a short timeframe is not directly comparable to long-term ethnographic research, SF’s objective was to understand the aesthetic relationships between human and sheep unfamiliar to one another, including how vulnerability toward a visitor might trigger flight responses. The familiarity of sheep to the farmer was presented in stark contrast to the familiarity of sheep to artist (see Figure 5). In particular, animals which were suffering from lameness would approach the farmers but would keep further distance from the artist. SF reflected this issue in his work, including online artistic documentations of the farm visits.

**Figure 6.**
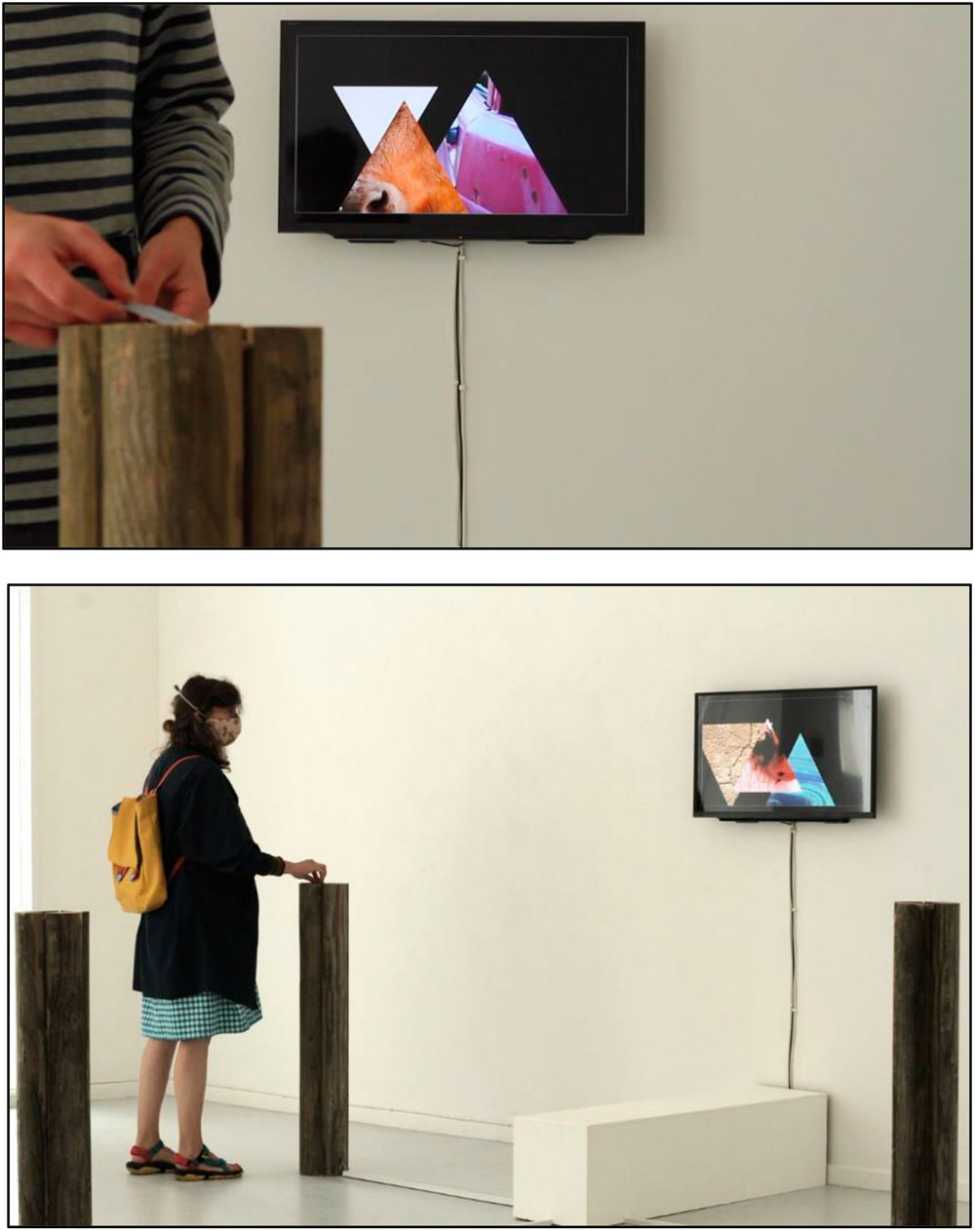
Shane Finan exhibition *It Seemed Like We Were Moving Closer Together* (2021). An interactive art installation exhibited at the Leitrim Sculpture Centre, Manorhamilton, Ireland, and the 19th Piksel Festival for Electronic Art and Free Technologies, Bergen, Norway (photographs from the former exhibition).

The types of farms visited by SF varied, including the ages and sizes of flocks and the practices of the farmers: the smallest flock was 40 sheep, the largest over 800. Yet there were consistencies: the farmers tracked and note the illnesses in their animals, and the artist was consistently an unfamiliar figure to the flocks and the farmers alike, inviting a guarded engagement. From this, SF considered vulnerability, guardedness, and distance as key topics in the aesthetic of the farm visits. The resultant work looked at touch and connection, considering how the human body and the sheep body are permanently apart from one another. In one artistic response, SF created artworks using the same radio frequency identification (RFID) technologies as found in microchips for recording sheep diseases and used footage and experience from field research to develop varied artworks. Incorporating a computer programme written by the artist and photographs taken on site visits, visitors were invited to scan RFID microchipped cards into RFID readers located inside fence posts. This changed images of animals and landscapes on a screen, creating a connection between the sensor, the image and the physical touch of a visitor and the visibility of the images of creatures on a screen (see Figure 6).

Using fence posts, images and RFID microchips, SF’s installation *It Seemed Like We Were Moving Closer Together* was one main culmination of the on-site research of FIELD. This artwork brought materials and behaviours into a non-farm space to consider how the bodily interaction (or lack of interaction) made a mark on a human audience. These artworks considered Puig de la Bellacasa’s question ‘can there be a detached touch?’, wherein she asks whether the disconnection of touching of bodies through technologies (mobile phones, medical equipment, RFID scanners) lowers the empathy between one human and another, or between a human and a nonhuman (Puig de la Bellacasa, 2017). Artistic research here is used both to complement the social science research and to illustrate some of the more complex ideas to a broader audience. SF chose this bodily approach because it helped to illustrate how boundaries are constructed not only around fields, but also within them, for example between farmer and animal. SF engaged with the corporeality of bodies, the artistic practice probing how bodies are enacted in relation to both other living organisms and the environments that they are moving in and through, contemplating too the marks made by infectious microorganisms on the bodies and body parts of sheep: for example, that these lifeforms may be present on one part of a body (e.g. the sheep’s foot) but that the marks of this presence may be observed in other body parts (e.g. the sheep’s head bobbing).

During the FIELD project a public engagement programme called ‘FIELDS’ was also undertaken. This included artist-led interventions at farm markets, video screenings and online presentations of documentation and artwork, presenting social scientific research alongside the artistic research of the AiRs. Artworks were presented as group and solo exhibitions in Ireland, Norway, Greece, and Slovakia. Visitors were invited to engage with the artworks and the artists, creating a community-engaged audience response that was framed by the art. Audiences of over 1,000 people attended these events, primarily in rural areas or small towns. While it is possible to quantify the number of visitors, it is difficult to document the quality of engagement, but anecdotally the artists were told stories about how their work had led people to think differently about their interactions with sheep. A further, important outcome of the AiRs’ work was the continued and sustained engagement between the artists and the researchers on the FIELD project, including the co-production of this paper.

## Conclusions

In this paper we explored, via social-scientific and artistic approaches, the presence of marks – as well as the variant of traces – on the places in which infectiousness is encountered on farms. We considered the ways in which these marks are made by infectious microorganisms on the bodies of farm animals, by farm animals themselves as they move across farmed spaces, and by humans when attempting to discover, make visible, and act upon the marks made by infectious microorganisms and farm animals.

Lifeforms (both farm animals and infectious microorganisms) moved in different ways and created different marks as evidence of this movement that required different responses from farmers and farm advisors. The marks included those made across the space of farms (e.g. the creation and use of tracks and paths) and on the spaces of bodies (e.g. the wounds and sores caused by infectious lameness), as well as marks made on the ways diseases were managed and on-farm biosecurity was enacted. Thus, marks could have physical, temporal, or metaphysical dimensions. In moving through farm spaces, farm animals and infectious microorganisms made a difference to the disease situations encountered by farmers and farm advisors, and to the management of farm animal disease. Animals often frustrated biosecurity practices of exclusion and enclosure (e.g. by crossing borders and boundaries) and were seen by the interviewees to exist on a spectrum of perceived disease risk (e.g. the disease risk of a farmer’s own, known animals compared to that of their neighbours’ unknown animals) (Hinchliffe et al., 2013; Hinchliffe & Bingham, 2008). Animals were thus indeed active ‘place-making agents’ (Bull & Holmberg, 2018, p.c2) co-creating the spaces of farms along with the humans that kept them and lived alongside them.

This paper also provides further evidence of the skilled and situated work performed by farmers and their advisors involved in the ‘patching together’ of on-farm biosecurity and disease management (Hinchliffe & Ward, 2014). The human actors – farmers especially – needed to learn to become attuned not only to the movements and typical behaviours of farm animals, but also to those of disease-causing infectious microorganisms, and how these groups of organisms might interact. I. In the case of sheep creating and producing pathways in a field, this behaviour might reduce the risk of parasitic worms, but increase the risk of infectious lameness, and farmers might need to respond to this complication in their management of the flock’s health. Human actors were therefore attentive to the many, different movements of animals and to the evidence of these movements on farms. This evidence presented as marks in the spaces of the farm, such as tracks, paths and transgressed borders, as well as in the physical composition of the bodies and body parts of farm animals, such as wounds, sores, and poorly growing bodies.

The artistic practice worked to illustrate farm animal bodies in motion and the marks made by this movement, illuminating the entangled relationships between infectious microorganisms, diseased animals and human actors on farms, showcasing how humans can become attentive to and transformed by these relationships. In so doing, the artistic output also focused viewers’ attention on to the corporeality of bodies and body parts by showing what lame sheep look like and how they behave. The artistic research allowed for a presentation of a more ethereal or embodied perspective without the burden of (conventional) data. The underlying research was thereby made secondary to the experience of a viewer who may take some learning about the movement of an animal body, or the boundary between animal and human, from the experience of an artwork.

Finally, the paper highlights the range of different other-than-human lifeforms involved in on-farm human-animal relationships, and particularly those relationships centred around the health of farm animals, including microorganisms that cause disease, such as viruses, existing ‘at the edge of life’. We suggest a continued focus on these limits of the animal – of animal geographies – in future social science research. For example, a greater focus on these organisms’ mobilities within farm spaces – to draw from the language of Hodgetts and Lorimer (2020) – even if, as ‘beings-in-relation’, these movements are facilitated by vectors such as other animals and objects. This optic is particularly timely given that the movements of some vectors are likely to change and become more prevalent under changing climate conditions. We also suggest a continued focus on the body parts involved in these relationships and how the sub-animal body contributes to managing disease on farms. This aspect has been illustrated here by interviewees when discussing the use of ear tag samples, which move and do work (e.g. they are transported to and tested in laboratories) far from the farm and the animal from which the flesh was removed. The work done by this body part then marks the (rest of the) animal that remains on the farm. It is also evidenced by the mentions of sub-animal fragments, such as cattle noses touching, in which parts, rather than bodies are crossing boundaries, and the structures of diseased sheep hooves. These matters and marks are important to consider when rethinking animal geographies.

## Notes

1 Mark Jones – https://vimeo.com/unmarkedfilms Michele Allen – https://www.michele-allen.co.uk Shane Finan – https://shanefinan.org

2 See: https://www.thomasthwaites.com/a-holiday-from-being-a-human-goatman/ for more details about the project.

3 Nine months is the length of the bovine gestation period.

4 To see the gif in motion please visit https://field.shanefinan.org/Images/02_fransje/animated_lameness_gif_sm.gif
